# Cell engineering with microfluidic squeezing preserves functionality of primary immune cells in vivo

**DOI:** 10.1073/pnas.1809671115

**Published:** 2018-10-31

**Authors:** Tia DiTommaso, Julie M. Cole, Luke Cassereau, Joshua A. Buggé, Jacquelyn L. Sikora Hanson, Devin T. Bridgen, Brittany D. Stokes, Scott M. Loughhead, Bruce A. Beutel, Jonathan B. Gilbert, Kathrin Nussbaum, Antonio Sorrentino, Janine Toggweiler, Tobias Schmidt, Gabor Gyuelveszi, Howard Bernstein, Armon Sharei

**Affiliations:** ^a^SQZ Biotechnologies, Watertown, MA 02472;; ^b^Oncology Discovery Translational Area, Roche Pharma Research and Early Development, Roche Innovation Center Zurich, 8952 Schlieren, Switzerland

**Keywords:** intracellular delivery, T cell engineering, microfluidics, cell therapy

## Abstract

The translational potential of cell-based therapies is often limited by complications related to effectively engineering and manufacturing functional cells. While the use of electroporation is widespread, the impact of electroporation on cell state and function has yet to be fully characterized. Here, we use a genome-wide approach to study optimized electroporation treatment and identify striking disruptions in the expression profiles of key functional transcripts of human T cells. These genetic disruptions result in concomitant perturbation of cytokine secretion including a 648-fold increase in IL-2 secretion (*P* < 0.01) and a 30-fold increase in IFN-γ secretion (*P* < 0.05). Ultimately, the effects at the transcript and protein level resulted in functional deficiencies in vivo, with electroporated T cells failing to demonstrate sustained antigen-specific effector responses when subjected to immunological challenge. In contrast, cells subjected to a mechanical membrane disruption-based delivery mechanism, cell squeezing, had minimal aberrant transcriptional responses [0% of filtered genes misregulated, false discovery rate (FDR) q < 0.1] relative to electroporation (17% of genes misregulated, FDR q < 0.1) and showed undiminished effector responses, homing capabilities, and therapeutic potential in vivo. In a direct comparison of functionality, T cells edited for PD-1 via electroporation failed to distinguish from untreated controls in a therapeutic tumor model, while T cells edited with similar efficiency via cell squeezing demonstrated the expected tumor-killing advantage. This work demonstrates that the delivery mechanism used to insert biomolecules affects functionality and warrants further study.

## Significance

Ex vivo manipulation of primary cells is critical to the success of this emerging generation of cell-based therapies, such as chimeric antigen receptor T cells for the treatment of cancer and CRISPR for the correction of developmental diseases. However, the limitations of existing delivery approaches may dramatically restrict the impact of genetic engineering to study and treat disease. In this paper, we compared electroporation to a microfluidic membrane deformation technique termed “squeezing” and found that squeezed cells had dramatically fewer side effects than electroporation and gene expression profiles similar to those of unmanipulated cells. The significant differences in outcomes from the two techniques underscores the importance of understanding the impact of intracellular delivery methods on cell function for research and clinical applications.

Engineering the genomes of primary human cells has significant therapeutic potential, but clinical translation is limited by efficacy and safety considerations associated with current delivery technologies (1–5). For example, advances in genome editing and gene therapy have brought hope for the development of new therapeutics in areas such as T cell engineering (6), hematopoietic stem cell (HSC) therapies (7), and regenerative medicine (8). Many technologies have been developed to address the challenge of intracellular delivery, but each has some limitations. For example, viral vectors have enabled delivery of gene-altering material into cells, but the translational potential of some viral vectors is limited by the risk of integrating viral sequences into the genome (9–12). Newer generation adeno-associated viruses have improvements in safety, but limitations associated with cargo size make them incompatible with classical gene editing tools. Electroporation as a nonviral alternative to deliver gene-engineering material removes risks specifically associated with viral delivery, but the functional consequences of doing so have not been fully examined.

Cell engineering relies on making directed changes to cell phenotype while maintaining cell functionality. The rigorous characterization of cell function postdelivery is equally important to quantifying target material efficiency. For example, achieving high editing efficiency of CD34+ HSCs for the treatment of β-thalassemia (13) and sickle cell disease (14) is only useful if engraftment potential is maintained. Similarly, T cells may be engineered to better target specific antigens (15), but nonspecific functional consequences leading to severe side effects and decreased efficacy must be minimized. While delivery efficiency and viability are important success metrics for cell engineering, nonspecific and unintended changes to cell phenotype may adversely impact functional potential.

Electroporation is a commonly used tool to deliver exogenous material into cells for therapeutic purposes, but the consequences of electroporation-induced disruptions on global gene expression, cytokine production, lineage markers, and in vivo function have not been fully characterized, particularly in the context of primary cells for cell therapy (16, 17). This is especially true for large macromolecules typically used for cell therapy, such as CRISPR-Cas9 ribonucleoproteins (RNPs) [Cas9 protein precomplexed with guide RNA (gRNA)] or DNA (18). Evidence suggests that the electroporation-mediated transfer of large molecules is likely a multistep process involving the poration of the cells, electrophoretic embedding of the material into the membrane, and, finally, the migration through the cytosol to the nucleus (19–21). Consequently, electroporation protocols have been empirically developed with narrow constraints on cell state, handling, pretreatment, and posttreatment. For example, rest times pre- and postelectroporation extend the time that cells must be in culture, and extended ex vivo culture risks terminal differentiation and the loss of a proliferative phenotype for T cells and CD34+ HSCs (22, 23). While electroporation protocols enable the efficient delivery of some payloads, challenges associated with posttreatment mortality, loss of proliferative potential, and decreased potency have been reported for primary cell types.

To address the potential consequences and concerns associated with subjecting cells to electric fields, we characterized the impact of well-established electroporation treatments on a transcriptional, translational, and phenotypic level. We also compared electroporation to a newer-generation microfluidic system, cell squeezing (24–28). We selected two cell types that are common targets for gene engineering: HSCs and T cells. We characterized changes at the genetic level with full transcriptome microarrays, validated protein expression for some markers of interest, and assessed in vivo phenotype. We found that optimized electroporation treatments did not significantly alter viability but were associated with dysregulation of key genes, functional pathways, and disease markers. Moreover, our in vivo data show diminished functional activity of electroporated T cells. In contrast, squeezed cells were similar to controls in gene expression and in vivo phenotype. Furthermore, we demonstrate efficacy of squeeze-edited T cells in a tumor model without the need for ex vivo T cell activation or rest periods before adoptive cell transfer. This work provides a detailed comparative analysis of the molecular signature and downstream functional consequences of electroporation relative to a mechanical delivery system and examines the potential delivery-associated changes in cells.

## Results and Discussion

### Modification of Delivery Parameters Impacts Cargo Uptake.

**Fig. 1.**
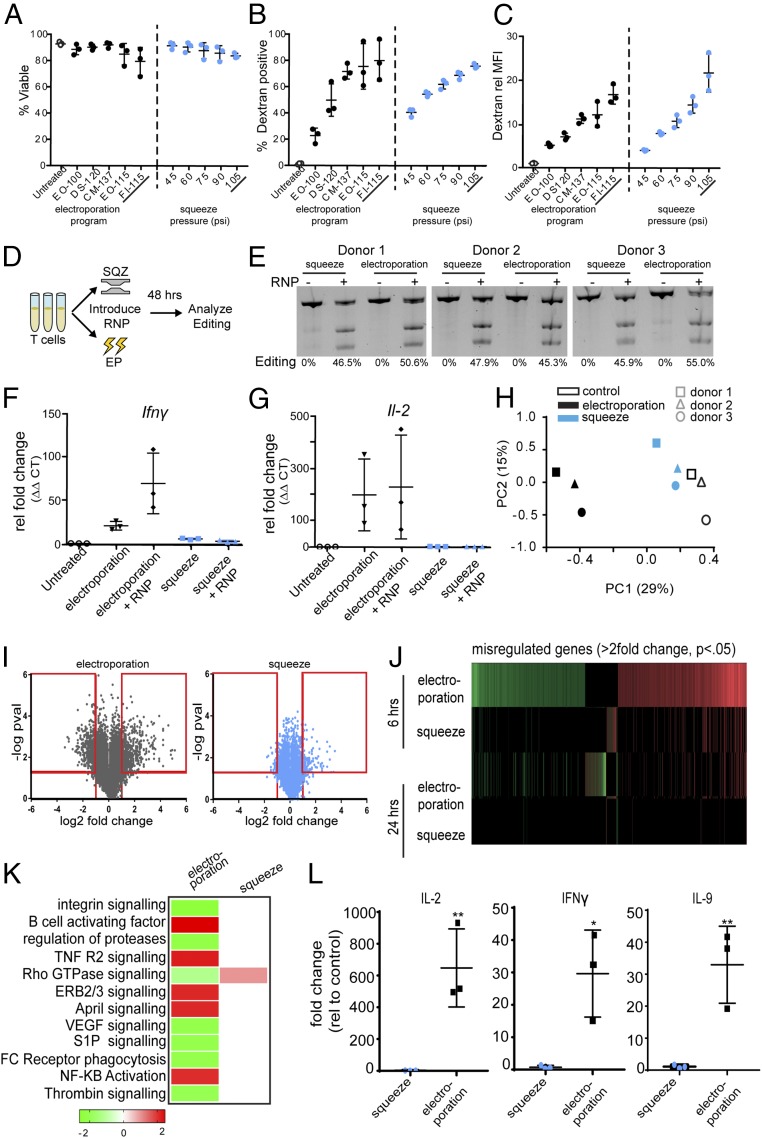
Comparison of intracellular delivery methods reveals delivery-mediated effects on gene expression and cytokine secretion in unstimulated human T cells. Screening electroporation programs (black) and cell-squeezing pressures (blue) reveal the impact of delivery protocol on (*A*) viability and (*B* and *C*) delivery. Delivery is represented as the total percent of cells (*B*) that received cargo (dextran) and the amount of cargo (*C*) that each cell received on a per-cell basis relative to untreated control (rel MFI). The conditions used for downstream editing and functional studies are underlined (electroporation program FI-115 and squeeze pressure 105 psi). (*D*) Graphical schematic detailing the workflow for the comparative editing studies. SQZ, squeeze; EP, electroporation. (*E*) T7E1 assay reveals gene editing efficiencies for squeeze and electroporation across three human donors. qPCR reveals *Ifnγ* (*F*) and *Il-2* (*G*) transcript levels in human T cells after electroporation ± RNP compared with untreated cells (*n* = 3 human donors). (*H*) PCA was performed across all T cell samples and all genes at the 6-h timepoint to generate a plot of PC1 (treatment variability) versus PC2 (donor variability). (*I*) Volcano plots show the fold change and *P* value of individual genes 6 h after electroporation (*Left*) and squeezing (*Right*) compared with controls (*n* = 3). The red boxes highlight genes with greater than twofold change and *P* value < 0.05 that were used in the (*J*) heat map and (*K*) pathway analysis (only pathways with a z-activation score >2 in either direction are shown). (*L*) Protein level validation of the array was done using cytokine secretion assays 24 h posttreatment (**P < 0.01; *P < 0.05).

The effects of intracellular delivery have not been fully described at the functional level, particularly in sensitive cell types such as unstimulated T cells. To this end, we set out to characterize the effects of electroporation on downstream cell function and to compare the results to a newer-generation microfluidic delivery system, cell squeezing. As a starting point, fluorescently tagged dextran was delivered to cells isolated from three human donors via several electroporation or cell-squeezing protocols, and analysis by flow cytometry was used to quantify delivery and viability at 6 h posttreatment. Electroporation test conditions were based on the manufacturer’s (Lonza) recommended unstimulated T cell protocols (FI-115 and EO-115) and published protocols (29) (EO-100, DS-120, and CM-137). Similarly, five different cell-squeeze pressures (45, 60, 75, 90, and 105 psi) were also tested. All conditions screened for electroporation and cell squeezing resulted in average viabilities of greater than 80% with no statistically significant difference between any condition (Fig. 1*A*). Given the high viability, we chose protocols for both technologies that resulted in optimal delivery of the dextran material—electroporation program FI-115 [79.5 ± 15.4% cells delivered, 16.8 ± 2.2 relative median fluorescence intensity (rMFI)] and cell-squeezing pressure 105 psi (75.3 ± 1.8% cells delivered, 21.7 ± 4.4 rMFI) (Fig. 1 *B* and *C*). Notably, the electroporation program we identified in this optimization screen is the same T cell-optimized electroporation program that is recommended by Lonza for high-efficiency delivery to unstimulated human T cells.

### Electroporation and Cell Squeezing Efficiently Edit Unstimulated Human T Cells.

The optimized protocols showed similar dextran delivery profiles for both technologies. To directly compare functional efficiency, we delivered Cas9 protein–gRNA RNP complexes (Cas9 RNPs) designed to target PD-1 via both technologies and assessed editing efficiency (Fig. 1*D*). Cas9 RNPs provide an attractive approach for engineering the genomes of primary human cells, and recent studies have reported efficient gene editing using electroporation as a means to deliver Cas9 RNPs to unstimulated T cells (29). We observed that the optimized squeeze (105 psi) and electroporation (FI-115) methods resulted in edited unstimulated T cells, and we found no statistically significant difference in editing efficiency between the two technologies (46.7 ± 0.9% vs. 50.3 ± 4.0%, respectively; *P* = 0.28) (Fig. 1*E*).

### Intracellular Delivery of Editing Reagents to T Cells Results in Nonspecific Up-Regulation of *Il-2* and *Ifnγ*.

To gain insight into the potential functional effects of delivery, we selected two genes that are critical for T cell function, *Ifnγ* (30) and *Il-2* (31), and used qPCR to characterize their expression levels after treatment with the delivery- and viability-optimized electroporation and cell-squeeze protocols. We found that electroporation, in the absence of RNPs, caused a 21-fold increase (*P* = 0.0021) in *Ifnγ* expression and a 196-fold increase (*P* = 0.06) in *Il*-*2* expression compared with untreated controls. The expression increases were further amplified when electroporation was done in the presence of RNPs, with *Ifnγ* and *Il-2* transcripts increasing 69-fold (*P* = 0.0265) and 226-fold (*P* = 0.1163), respectively (Fig. 1 *F* and *G*). We found that squeezing cells had dramatically fewer side effects on *Ifnγ* and *Il-2* transcript levels (twofold, *P* = 0.0247 and sixfold, *P* = 0.0085, respectively) with no significant difference when done in the presence of PD-1 targeting RNP (Fig. 1 *F* and *G*). Taken together, these results suggest that intracellular delivery mechanisms may cause unintended and nonspecific changes to cell function.

### Genome-Wide Profiling Reveals the Impact of Intracellular Delivery on Baseline Gene Expression.

The changes in *Ifnγ* and *Il*-*2* transcripts prompted us to examine the impact of electroporation and cell squeezing on a genome-wide scale. Given that the mechanisms by which squeezing and electroporation mediate intracellular delivery differ, we reasoned that the downstream consequences of delivery may also be different between the two technologies. Gene expression profiling has been used to de-risk therapeutic agents under development in major drug categories, such as small molecules and biologics, but such methods have not been applied to electroporation transfection technology despite its use in therapeutics. Using the optimized conditions, we treated unstimulated T cells with electroporation or squeezing and analyzed the cells with an unbiased and comprehensive genome-wide microarray approach at 6 and 24 h posttreatment. We also assessed viability via flow cytometry at the 24-h timepoint and found slight decreases in viability for all groups, regardless of treatment, at 24 h (81.3 ± 8.4% untreated, 70.2 ± 7.2% squeeze, 62.6 ± 5.1% electroporation). Cells were treated in the absence of cargo because our qPCR results (Fig. 1 *F* and *G*) showed that the inclusion of RNPs exacerbated electroporation-induced misexpression, suggesting that the cargo may confound the results of a genome-wide study comparing delivery methods.

Despite the maintenance of viability posttreatment, significant and dramatic changes in gene expression were associated with electroporation 6 h posttreatment. We calculated FDR q values on filtered gene sets to determine the number of genes that were misexpressed after treatment with squeeze or electroporation compared with the untreated controls. In primary human T cells we found that 34% of all genes were misexpressed after electroporation (8,141/23,786, FDR q < 0.25), while 9% of genes were misexpressed after squeezing (2,211/23,786, FDR q < 0.25) (Table 1 and Dataset S1). Using a more stringent FDR cutoff of 0.1, we found that electroporation resulted in 17.1% (4,072/23,786) of genes misexpressed versus 0% (0/23,786) in squeezed samples (Table 1 and Dataset S1). To further compare the effects of the various treatment methods across all genes, a principal component analysis (PCA) was conducted. The PCA showed electroporated samples separating away from squeezed and unmanipulated controls along the PC1 axis, suggesting that electroporation is the largest driver of variance in gene expression (29% variance across the PC1 axis; Fig. 1*H*). We performed a transcriptome microarray analysis similar to that described above using CD34+ HSCs that were treated with the manufacturer’s recommended electroporation program (EO-100) for CD34+ HSCs and observed similar patterns in viability and PCA analyses (*SI Appendix*, Fig. S1 *A* and *B* and Dataset S2). The significant changes in gene expression across both primary cell types demonstrate the profound impact that electroporation can have on gene expression.

**Table 1.** FDR q-value thresholds for pairwise comparisons in unstimulated T cells

	6 h		24 h	
q threshold	Electroporation vs. control	Squeeze vs. control	Electroporation vs. control	Squeeze vs. control
0.25	8,141	2,211	3,309	0
0.1	4,072	0	7	0
0.05	330	0	1	0

### Post Hoc Analysis of Gene Expression Profiles Differentiates Delivery Approaches.

Based on the 23,786 gene probes surveyed in the microarray analysis, we generated volcano plots comparing the effect of treatment to controls and again found evidence of the dramatic impact that electroporation has on gene expression in T cells (Fig. 1*I*) and CD34+ HSCs (*SI Appendix*, Fig. S1*C*). Next, we narrowed the gene sets to include only those genes that were dramatically affected by applying a filtering criterion of greater than twofold change (*P* < 0.05) relative to untreated controls (Fig. 1*I*, red boxes). Using this filtered gene set comprising 1,123 genes in the electroporated T cells and 147 genes in the squeezed T cells, we generated a heat map (Fig. 1*J*) and completed an ingenuity pathway analysis which identified 120 canonical pathways affected by electroporation compared with only one canonical pathway affected by squeezing (Dataset S3). Pathways with a directional activation z-score of >2 in either direction are shown (Fig. 1*K*). The single pathway affected by squeeze, also affected by electroporation, involves signaling by Rho family GTPases. This result is consistent with a role for members of the Rho GTPase family in many aspects of intracellular actin and cytoskeletal dynamics (32) which are affected by the deformation of the membrane. The diversity of misregulated pathways related to T cell survival, activation, and DNA damage postelectroporation was unexpected, especially given the maintenance of viability, and suggests that electroporation impacts multiple cell functions, including nonspecific activation and DNA damage responses.

To examine the kinetics of gene expression we compared the expression of misregulated genes at 6 and 24 h for T cells (Fig. 1*J* and Dataset S1) and CD34+ HSCs (*SI Appendix*, Fig. S1*D* and Dataset S2). Using the same filtering criterion (greater than twofold change, *P* < 0.05) we found that the initial misexpression signature of electroporated T cells was reduced after 24 h in culture (Fig. 1*J*), indicating that some genes were returning to basal expression levels. However, this comparison revealed a subset of genes in the electroporated cells that were newly affected after 24 h in culture for both T cells (Fig. 1*J* and Dataset S1) and CD34+ HSCs (*SI Appendix*, Fig. S1*D* and Dataset S2), suggesting knock-on effects in response to electroporation over time.

Closer inspection of the individual genes linked to activation and stress response pathways showed that electroporated T cells dramatically up-regulated cytokines including *Ifnγ* (29.3-fold, *P* < 0.01), *Cxcl9* (18.6-fold, *P* < 0.05), *Cxcl10* (14.9-fold, *P* < 0.01), and *Il-2* (14.7-fold, *P* < 0.05) compared with untreated control cells (Dataset S1). Conversely, cells subjected to squeezing did not show these increases. Importantly, the misregulation of functional genes and pathways highlights the risk of electroporation confounding research conclusions and limiting therapeutic efficacy. Taken together, these data suggest the need for a more rigorous characterization of cell phenotype and function posttreatment.

### Electroporation Causes Broad Cytokine Secretion in Human T Cells.

The gene sets that we identified at the transcript level prompted us to further characterize the secretion profiles of key cytokines in T cells. As with the microarray, we treated T cells from three human donors in the absence of cargo with squeeze or electroporation. After 24 h in culture, the supernatants were collected for a multiplex cytokine panel to determine if the changes we observed at the genetic level resulted in changes in cytokine secretion. In response to electroporation, we identified significant and striking increases in secretion of key functional cytokines, including IL-2 (648-fold increase, *P* < 0.01), IL-9 (33-fold increase, *P* < 0.01), IFN-γ (30-fold increase, *P* < 0.05), and TNF-α (10-fold increase, *P* < 0.05) (Fig. 1*L* and Dataset S4). Consistent with the microarray, there were minimal consequences to cytokine secretion in response to squeezing, with none of the cytokines assayed showing statically significant differences in secretion compared with untreated control (Dataset S4). In addition, 31% (13/42) of the cytokines assayed showed significantly different expression profiles in response to electroporation compared with squeeze and control (Dataset S4). Notably, many of the cytokines that were up-regulated by electroporation (IL-2, IFN-γ, TNF-α, IL-6, IL-8, and IL-10) have known roles in toxicological pathways associated with cytokine release syndrome (33). The release of cytokines from engineered cells, such as chimeric antigen receptor T cells, is a common and sometimes lethal complication, and our data suggest that electroporation, even in the absence of target engagement, increases the nonspecific release of cytokines.

To confirm that these results were not atypical of squeeze and electroporation conditions, we conducted cytokine analyses on multiple additional conditions from the original optimization screen (Fig. 1 *A*–*C*). Our results showed that even the potentially “gentler” electroporation protocol (EO-100), as inferred by lower delivery (22.8 ± 5.6% of live cells receiving dextran, 5.183 ± 0.431 rMFI) (Fig. 1 *B* and *C*), was capable of inducing significant cytokine misregulation, while the squeeze conditions consistently showed minimal change (*SI Appendix*, Fig. S2). This indicates that across both technologies delivery efficiency is decoupled from functional side effects—highlighting the importance of assessing and optimizing for function independently.

### Exposure to Electroporation Decreases Colony-Forming Potential of CD34+ HSCs.

**Fig. 2.**
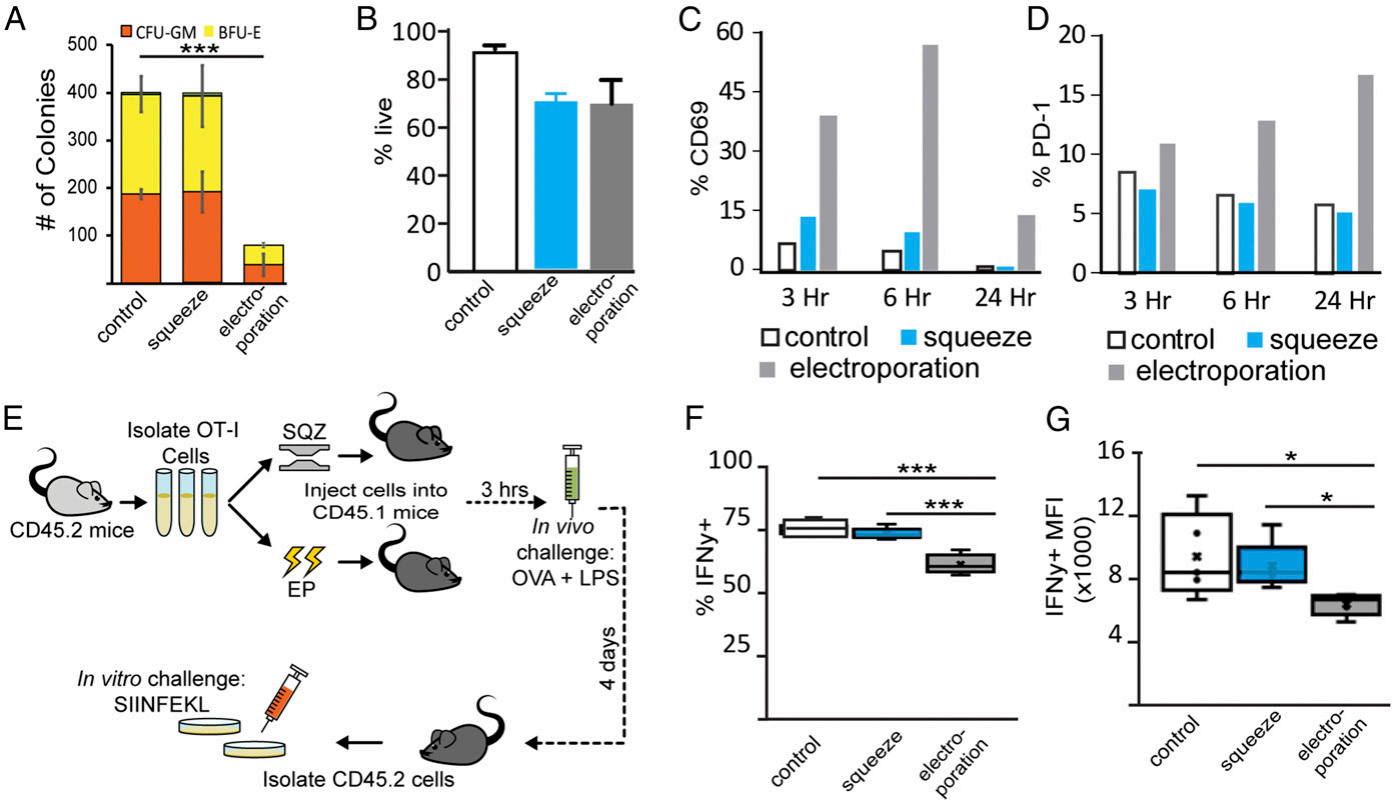
Intracellular delivery methods impact in vitro and in vivo functionality. (*A*) In vitro colony-forming assays compare the potential of electroporated and squeezed human CD34+ HSCs to differentiate into colony-forming unit granulocyte–macrophage (CFU-GM) and burst-forming unit erythroid (BFU-E) colonies over the course of 2 wk. (*B*) Viability of murine T cells after squeeze and electroporation is shown. (*C* and *D*) Representative percentage of CD3+ murine T cells that exhibit PD-1 or CD69 activation after squeeze, electroporation, or no treatment (control) over time is shown. (*E*) A schematic detailing the experimental approach to assess delivery-mediated effects on T cell activation is shown. (*F* and *G*) After T cell rechallenge with OVA on day 4, CD45.2+/CD8+/IFN-γ+ T cells were intracellularly stained for IFN-γ (***P ≤ 0.001; *P < 0.05).

To determine if the transcript-level changes postelectroporation or postsqueeze resulted in functional defects, we tested the ability of CD34+ HSCs to differentiate into specific lineages in vitro immediately posttreatment. Consistent with the microarray results, in vitro colony-forming assays showed that squeezed cells performed similarly to untreated control cells, producing comparable numbers and types of colonies. Both the control and squeezed cells significantly outperformed electroporated cells, each forming more than four times more colonies than the electroporated cells (*P* ≤ 0.001, Fig. 2*A*). Throughout the course of the 2-wk-long assay, the electroporated cells lagged behind the control and squeezed cells. Additional studies need to be done to determine if the reduced colony-forming potential postelectroporation is the result of long-term functional side effects or transient delays in initial proliferation postelectroporation.

### Murine T Cells Are Subject to Delivery-Mediated Phenotypes Similar to Human T Cells.

To assess potential consequences in vivo, we conducted murine studies using the same squeeze conditions optimized for human T cells and the manufacturer’s recommended electroporation protocol for unstimulated murine T cells (DN-100). We sought to determine if treatment-mediated T cell perturbation resulted in changes to murine T cell phenotype in a manner similar to that demonstrated in the human T cells. Given that the human cell studies showed evidence of nonspecific activation postelectroporation, we assessed expression trends of two common activation markers, CD69 and PD-1, posttreatment via flow cytometry over time. We again found high viability posttreatment with both technologies (Fig. 2*B*); however, measurement of CD69 and PD-1 surface activation markers revealed higher surface-level expression in electroporated murine T cells than in squeezed cells and controls at each timepoint (Fig. 2 *C* and *D* and *SI Appendix*, Fig. S3 *A* and *B*). CD69 activation due to electroporation appeared transient as CD69 levels decreased over time (Fig. 2*C* and *SI Appendix*, Fig. S3*A*). However, electroporation-mediated PD-1 up-regulation persisted with increasing PD-1 levels over time (Fig. 2*D* and *SI Appendix*, Fig. S3*B*), suggesting that electroporated T cells were entering an exhausted state.

### In Vivo Challenge of Murine T Cells Reveals That Delivery-Mediated Side Effects Can Persist for Several Days.

Since increased PD-1 levels have been linked to anergic CD8+ T cell responses (34), we used an in vivo antigen challenge assay to test if there were lasting consequences to T cell effector function after treatment (Fig. 2*E*). We confirmed that the electroporation and squeeze treatments resulted in high viability and delivery using a dextran tracer molecule (*SI Appendix*, Fig. S3*C*). T cells from congenically marked CD45.2 OT-I transgenic mice were electroporated or squeezed without cargo and adoptively transferred into CD45.1 congenic mice. Three hours posttransfer, mice were challenged with an s.c. injection of ovalbumin (OVA) and an adjuvant to induce antigen-specific activation. Four days postinjection, T cells were collected from the draining lymph nodes and rechallenged ex vivo with the OVA minimal epitope SIINFEKL peptide. Electroporation of T cells resulted in a reduction of the percentage of IFN-γ+ T cells (Fig. 2*F* and *SI Appendix*, Fig. S3*D*) and the expression of IFN-γ as measured by MFI of IFN-γ+ cells (Fig. 2*G* and *SI Appendix*, Fig. S3*D*). Conversely, there was no significant difference in either measurement of IFN-γ between squeezed cells and control cells (Fig. 2 *F* and *G*).

Taken together, the significant increase in cytokine secretion of electroporated human T cells and the blunted IFN-γ response after antigen-specific challenge in electroporated murine T cells suggests that electroporation causes nonspecific cytokine bursts immediately posttreatment that cannot be sustained and ultimately dampen effector function long-term. Specifically, these data suggest that electroporation induces suboptimal activation that in turn reinforces anergic signals in transplanted CD8+ T cells. Nonspecific activation followed by diminished antigen-specific effector function was not observed in the squeezed or control T cells. These data suggest an electroporation-mediated dampening of T cell effector function that may ultimately limit the efficacy of therapies based on electroporated T cells.

### Squeeze-Edited T Cells Show Efficacy in Tumor Models.

**Fig. 3.**
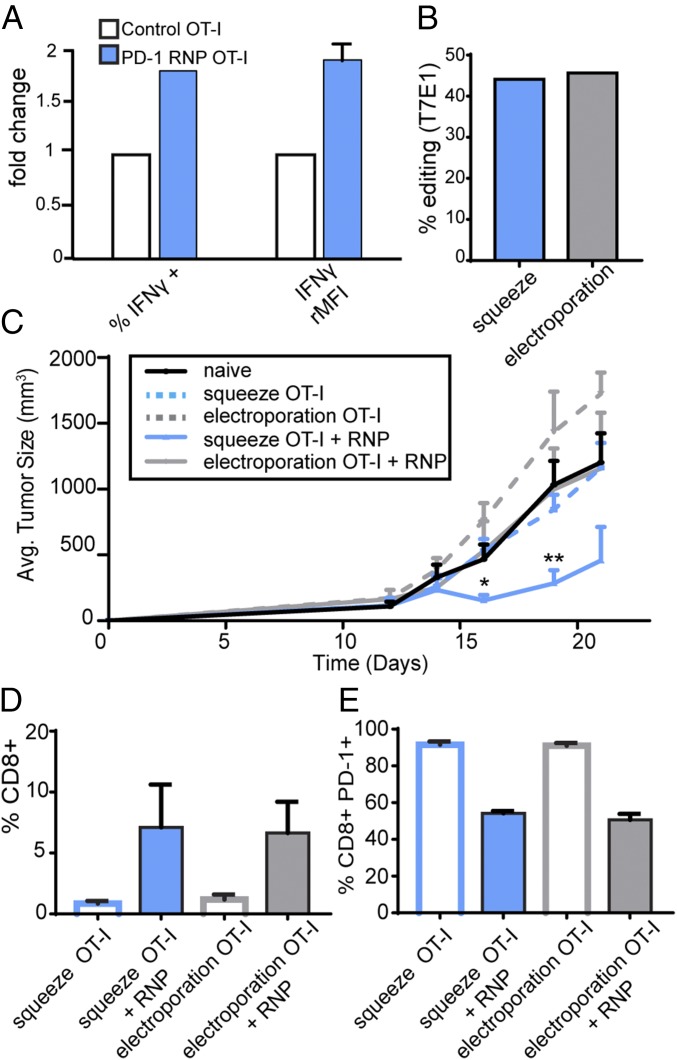
Squeeze-edited T cells demonstrate efficacy in the tumor setting. (*A*) ICS results show the percent of IFN-γ–positive cells (*Left*) and rMFI of IFN-γ (*Right*) in squeeze-edited and control murine OT-I T cells when cocultured with B16-OVA tumor cells. (*B*) A subset of edited OT-I T cells were cultured ex vivo to determine the input editing efficiency for both squeeze and electroporation in the tumor study. (*C*) Tumor growth measurements compare therapeutic administration of untreated wild-type OT-I T cells (naïve) to wild-type OT-I T cells treated with squeeze or electroporation, and squeeze-edited or electroporation-edited OT-I T cells (*n* = 5 animals per group; **P < 0.01; *P < 0.05; comparisons were done relative to the naïve group). (*D*) TIL analysis quantifying percent of CD8+ CD45.2+ cells that have infiltrated the tumor is shown. (*E*) TIL analysis was done to quantify the percent PD-1+, CD45.2+, CD8+, OVA Tetramer+ cells infiltrating the tumor.

Next, we tested the therapeutic potential of squeeze-edited cells in the tumor setting. We leveraged the murine OT-I system and designed a series of experiments to determine if PD-1 edited T cells phenocopy antibody-mediated PD-1 blockade by assessing their effector function in vitro and in vivo. First, we identified gRNA pairs targeting murine PD-1 and tested their activity in vitro. The edited T cells were then activated and cocultured with an OVA-expressing syngeneic tumor cell line. IFN-γ levels were measured by intracellular cytokine staining (ICS), revealing a 79% increase in IFN-γ–positive cells and a 90% increase in IFN-γ MFI in the edited T cells compared with wild-type controls (Fig. 3*A*). These results suggest enhanced effector function with squeeze-mediated engineering of PD-1.

To directly compare effector function of our squeeze-engineered PD-1 knockout T cells to those engineered with electroporation, we used an in vivo syngeneic tumor model. Unstimulated splenic OT-I T cells (CD45.2) were isolated and squeezed or electroporated with PD-1 RNP, and equivalent numbers of live cells were immediately transferred into CD45.1 mice bearing EG.7 OVA tumors (average preadoptive transfer tumor size = 200 mm^3^). A small subset of cells was removed before adoptive cell transfer to confirm comparable editing efficiency between the two technologies (Fig. 3*B*). Tumor growth monitoring revealed that only squeeze-edited cells were able to control tumor growth (average tumor size = 154.8 mm^3^ ± 42.8 squeeze OT-I + RNP vs. 466.7 mm^3^ ± 112.8 naïve at day 16, *P* < 0.05; 458 mm^3^ ± 254.9 squeeze OT-I + RNP vs. 1202 mm^3^ ± 222.5 naïve at day 19, *P* < 0.05), whereas electroporation-edited cells, despite having similar editing efficiency, were unable to control the tumors (Fig. 3*C* and *SI Appendix*, Fig. S3*E*). Analysis of the tumor-infiltrating lymphocytes (TILs) revealed similar amounts of CD8+ T cell infiltrate into the tumors and similar PD-1 levels between squeeze and electroporation (Fig. 3 *D* and *E*). Despite the similar infiltration levels and editing efficiencies, the inability of electroporated-edited cells to control tumor growth relative to squeeze-edited cells suggests that electroporated cells lack full functionality, thereby prohibiting them from eliciting desired phenotypes in the therapeutic setting in vivo. Taken together, these results highlight that, despite successful genetic alteration (in this case PD-1 deletion), delivery-method-induced alterations may prevent the desired biological effect in the target cells.

## Conclusions

Concerns surrounding efficacy and safety of different cell engineering technologies have limited cell-based therapies in the past. As such, characterizing the effect of delivery systems on the functional level, in addition to delivery and viability, is important. While recent studies have focused on optimizing delivery and viability with electroporation (29), they have failed to fully characterize the downstream functional consequences. Genome-wide expression profiling techniques, such as microarrays, have the capacity to detect comprehensive transcriptomic alterations in the target cells. We leveraged these tools to reveal significant, unintended consequences related to delivery mechanism. We show that dramatic, albeit transient, disruption at the transcript level driven by electroporation can have long-lasting functional effects. While both electroporation and cell squeezing rely on membrane disruption to some extent, our work suggests that mechanical membrane disruption coupled with diffusion-mediated delivery dramatically reduces unintended negative consequences. As illustrated by the described antitumor (Fig. 3*C*) and cell differentiation studies (Fig. 2*A*), critical biological attributes of the engineered cells could be lost as a result of the selected delivery strategy. Hence, for both research and therapeutic applications, the functional and safety consequences of the selected intracellular delivery technique and its impact on cell phenotype should be carefully evaluated.

## Materials and Methods

### Ethics Statement.

All of the experimental methods were carried out in accordance with the approved guidelines. The blood collection procedure was carried out in accordance with guidelines approved by the New England Independent Review Board. All donors signed an informed consent for scientific research statement. All animal work was carried out under protocol 201704-AVS in accordance with guidelines approved by the Institutional Animal Care and Use Committee, the Public Health Service Policy on Humane Care and Use of Laboratory Animals, and the US Government Principles for Utilization and Care of Vertebrate Animals used in Testing, Research and Training.

### Cell Isolation.

Human peripheral blood mononuclear cells (PBMCs) were isolated from fresh blood using Ficoll gradient centrifugation, and T cells were isolated using StemCell EasySep Human T cell enrichment Kit (catalog no. 19051) according to the manufacturer’s protocol. Murine T cells were isolated directly from the spleen and lymph nodes using StemCell EasySep mouse T cell isolation kit (catalog no. 19851) according to the manufacturer’s protocol. Peripheral blood-derived CD34+ HSCs were thawed using standard methods, cultured overnight, then treated the following day.

### Cell Squeezing (CellSqueeze) and Microfluidic Devices.

Cells were resuspended at 20 × 10^6^ million cells/mL in X-VIVO 10 (Q4-04-744Q; Lonza) as the delivery buffer. Cell squeezing was done using previously established methods (24, 25) with the following specifications. When appropriate, cargo was added to the cells and delivery buffer. The cells were squeezed through microfluidic channels containing a single 3.5-µm-wide, 30-µm-long constriction. In the functional studies, T cells were squeezed at 105 psi, whereas HSCs were squeezed at 90 psi.

### Electroporation.

Cells were electroporated with the 4D-Nucleofector System (Lonza) (100-µL cuvette format) as per the manufacturer’s protocol and specified buffer (P3 Primary Cell 4D-Nucleofector Solution). Optimized, preloaded pulse programs for the specific cell type were used for functional studies (human T cells, FI-115; murine T cells, DN-100; human CD34+ cells, EO-100).

### Cell Culturing.

Human T cells were cultured in X VIVO-15 culture media (Lonza) supplemented with 5% human AB serum, 1% penicillin/streptomycin (pen/strep), and 5 ng/mL human IL-15 at 1–2 million cells per mL. Murine CD3+ T cells isolated from splenocytes were cultured in RPMI culture media supplemented with 10% FBS and 1% pen/strep, 100 U/mL IL-2, 20 ng/mL IL-7, and 10 ng/mL IL-15. CD34+ HSCs were cultured in X VIVO-10 with 1% pen/strep and 100 ng/mL of SCF, TPO, and Flt3L.

### Flow Cytometry Analysis.

Flow cytometry was performed using Attune NxT (Thermo Fisher). Data were examined using FlowJo.

### RNP Complexes.

Cas9 protein was delivered to a final concentration of 0.1 mg/mL for squeeze and electroporation. For each 100-µL reaction for both squeeze and electroporation, 10 µg of recombinant sNLS-spCas9-sNLS Cas9 nuclease (Aldevron 9212-PC) was precomplexed with a 2.5 molar excess of unmodified gRNA then delivered. Dual gRNAs were used to target the human and murine PD-1 loci (human PD-1 targeting gRNA: GCAGTTGTGTGACACGGAAG and GCGTGACTTCCACATGAGCG, murine PD-1 targeting gRNA: AGTTGAGCTGGCAATCAGGG and TGAATGACCAGGGTACCTGC).

### Microarray.

For both CD34+ HSCs and T cells, Human 2.0 Gene (Affymetrics) arrays comprising cells treated with electroporation, squeeze, or unmanipulated were done. Cells were harvested from three donors, and RNA was extracted from cells at 6 and 24 h (*n* = 3 per treatment group).

### Microarray Analysis.

Human 2.0 gene array CEL files were normalized to produce gene-level expression values using the implementation of the Robust Multiarray Average (RMA) in the affy package (Version 1.36.1) included in the Bioconductor software suite (Version 2.12) and an Entrez Gene-specific gene mapping (17.0.0) from the Molecular and Behavioral Neuroscience Institute (Brainarray) at the University of Michigan. Array quality was assessed by computing Relative Log Expression (RLE) and Normalized Unscaled SE (NUSE) using the affyPLM package (Version 1.34.0). PCA was performed using the prcomp R function with expression values that had been normalized across all samples to a mean of zero and an SD of one. Linear mixed-effects modeling and the associated analysis of variance were carried out using the anova.lme function in the nlme package (Version 3.1-97). Correction for multiple hypothesis testing was accomplished using the Benjamini–Hochberg FDR. All microarray analyses were performed using the R environment for statistical computing (Version 2.15.1).

### Multiplex Assays.

For each multiplex experiment, T cells were isolated from PBMCs from three human donors. Cells were treated in the absence of cargo. Live cells counts were determined via flow cytometry, and cells were cultured for 24 h at a density of 2 × 10^6^ cells/mL in X VIVO-15 (Lonza) supplemented with 2 mM l-glutamine and 5% AB serum.

### Colony-Forming Cell Assay.

CD34+ HSCs were squeezed or electroporated. Samples were counted, and viability was checked via flow cytometry immediately posttreatment. Cells were plated in six well plates at 1,500 live cells per well with MethoCult (SC 4435). Two weeks later colonies were counted.

### In Vitro Murine T Cell Activation Assay.

CD3+ T cells were isolated from the spleen and lymph nodes of wild-type C57BL/6J mice. Isolated cells were squeezed, electroporated, or incubated (control) with 3 kDa dextran. Samples were stained with PD-1 or CD69 fluorescent surface markers to assess T cell activation 3 h, 6 h, or 24 h after dextran delivery. Marker-specific activation was measured with flow cytometry.

### In Vivo Murine T Cell Activation Assay and ex Vivo Restimulation.

CD3+ T cells were isolated from the spleen and lymph nodes of OT-I transgenic C57BL/6J mice. Isolated cells were squeezed, electroporated, or untreated (control) and then immediately adoptively transferred via retroorbital injection into host CD45.1 transgenic C57BL/6J mice at 4 million live OT-I T cells per mouse. Three hours after OT-I T cell injection, host CD45.1 mice received 200-µL s.c. injections (100 µL into each flank) containing in total 10 µg SIINFEKL peptide (Anaspec), 1,000 U IL-2 (Peprotech), and 25 µg ODN 1826 (Invivogen) resuspended in Sigma adjuvant emulsion (Sigma-Aldrich). Four days later draining inguinal lymph nodes were removed for intracellular staining. Cells isolated from lymph nodes were seeded at 2 million cells per well in a 96-well U-bottom plate and cultured in RPMI + 10% FBS + 2 µg/mL anti-mouse CD28 (BD biosciences) ±1 µg/mL SIINFEKL or ± 1× Ionomyocin/PMA (positive control) at 37 °C. One hour after the start of the culture golgi-plug and golgi-stop (BD) inhibitors were added followed by a 4-h incubation before FACS staining for CD45.1, CD45.2, CD8, and IFN-γ.

### In Vivo Murine Therapeutic Study with PD-1 Edited Cells.

CD45.1 mice were inoculated with 10^5^ E.G7 OVA tumor cells s.c. and monitored for tumor growth until tumor average size reached 0.2000 mm^3^. Mice grouped to create an equal distribution of tumor size across the groups (groups: naïve, squeeze OT-I, electroporation OT-I, squeeze OT-I + RNP, and electroporation OT-I + RNP) with five animals per group. Treated (as described above) and control OT-I T cells (CD45.2) were transferred into tumor-bearing CD45.1 mice via retroorbital injection. Tumor growth monitoring ensued until untreated control mice reached the humane endpoint (1,500 mm^3^ tumor burden or 20% loss in net body weight). At the terminal time point, TIL analysis via FACS was performed. Briefly, tumors were extracted and dissociated with the GentleMax dissociator and Tumor Dissociation enzyme mix (130-096-730) as per the manufacturer’s recommendations. Cells underwent ICS/FACS staining using CD8a (53-6.7; BD Biosciences), CD45.1 (A20; BioLegend), PD-1 (RMP1-30; BioLegend), CD4 (GK1.4; BioLegend), CD45.2 (104; BioLegend), and Live/Dead (Near-IR; Thermo Fisher Scientific) stains.

## Supplementary Material

Supplementary File

Supplementary File

Supplementary File

Supplementary File

Supplementary File
