# Supplementary material for: Cell engineering with microfluidic squeezing preserves functionality of primary immune cells in vivo
Source: Proc Natl Acad Sci U S A. 2018 Oct 31;115(46):E10907–14. doi: 10.1073/pnas.1809671115 (PMC6243275; doi:10.1073/pnas.1809671115)
Supplement: Supplementary File [file pnas.1809671115.sapp.pdf]

PNAS
www.pnas.org

Supplementary Information for

Cell engineering with microfluidic squeezing preserves functionality of primary immune cells *in vivo*

Tia DiTommaso[1], Julie M Cole[1], Luke Cassereau[1], Joshua A Buggé[1], Jacquelyn L Sikora Hanson[1], Devin T Bridgen[1], Brittany D Stokes[1], Scott M Loughhead[1], Bruce A Beutel[1], Jonathan Gilbert[1], Kathrin Nussbaum[2], Antonio Sorrentino[2], Janine Toggweiler[2], Tobias Schmidt[2], Gabor Gyuelveszi[2], Howard Bernstein[1], Armon Sharei[1]

[1] SQZ Biotech, Watertown, USA
[2] Roche Pharma Research and Early Development, Oncology Discovery Translational Area, Roche Innovation Center Zurich, Schlieren, Switzerland

Corresponding Author: Tia DiTommaso, tditommaso@sqzbiotech.com

This PDF file includes:

Supplementary Figures 1-3

www.pnas.org/cgi/doi/10.1073/pnas.1809671115

Supplementary Figure 1

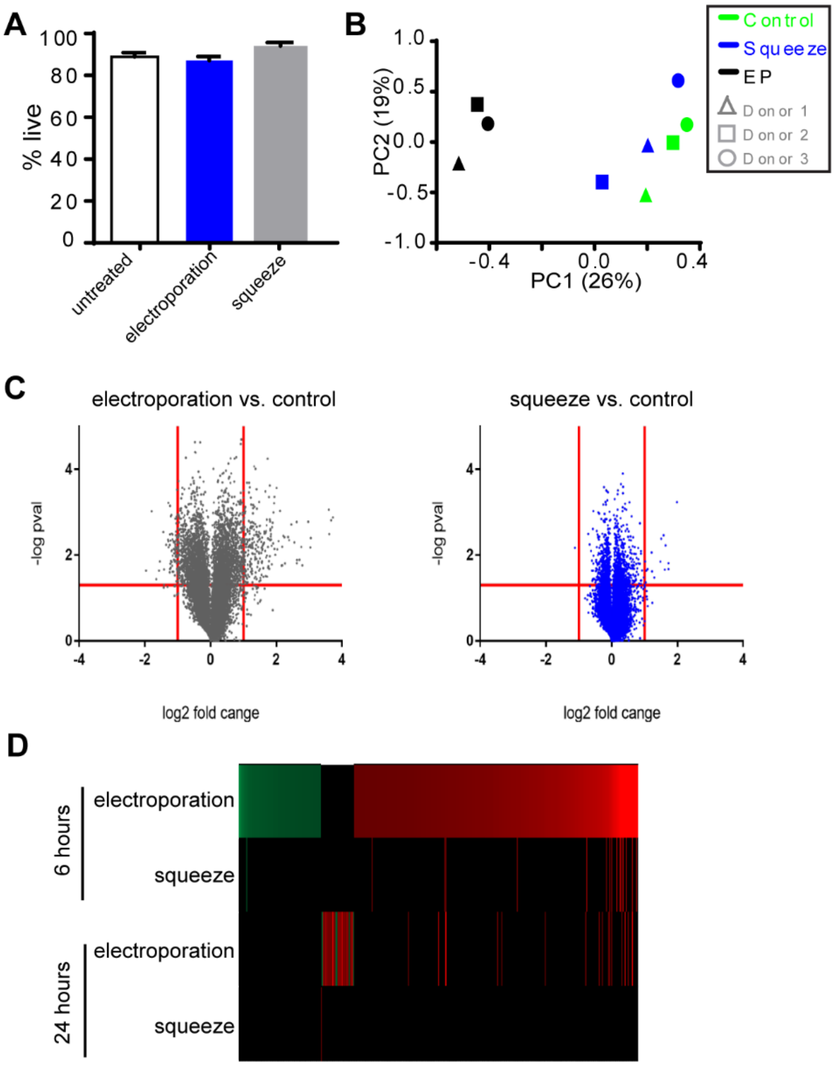

Supplementary Figure 1: Comparison of Cell Engineering Methods in CD34+ HSCs. A) Viability of CD34+ HSCs post-treatment was measured via FACS. **B)** PCA was performed across all genes and all samples in CD34+ HSCs, and a plot comparing PC1 vs. PC2 was generated. **C)** Volcano plots show the fold-change and significance of gene expression for treated cells as compared to controls. Red boxes indicate genes with >2-fold change ($p < 0.05$). **D)** A heatmap showing mis-regulated genes after filtering criteria of >2-fold change, $p < 0.05$.

Supplementary Figure 2

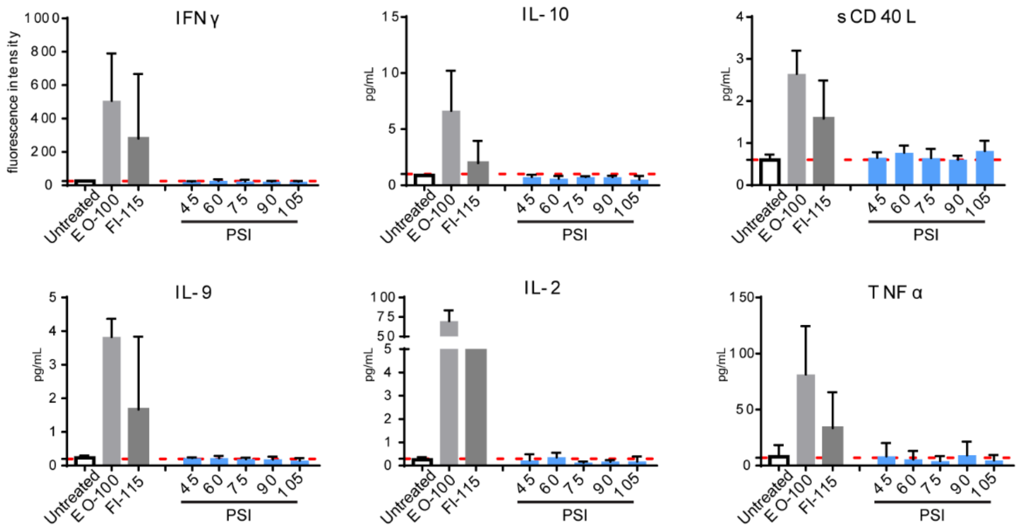

Supplementary Figure 2: Multiplex Comparison of Cytokine Profiles. Unstimulated human T cells were subjected to low delivery (EO-100) and high delivery (FI-115) electroporation protocols and a range of cell squeeze pressures to determine if delivery profile impacted cytokine secretion at 24-hours post-treatment. The fluorescence intensity (of IFNγ) or secretion (pg/mL, for IL-10, sCD40L, IL-9, IL-2, TNFα) were assessed. Error bars represent SD, dotted red line represents average cytokine secretion of untreated controls.

Supplementary Figure 3

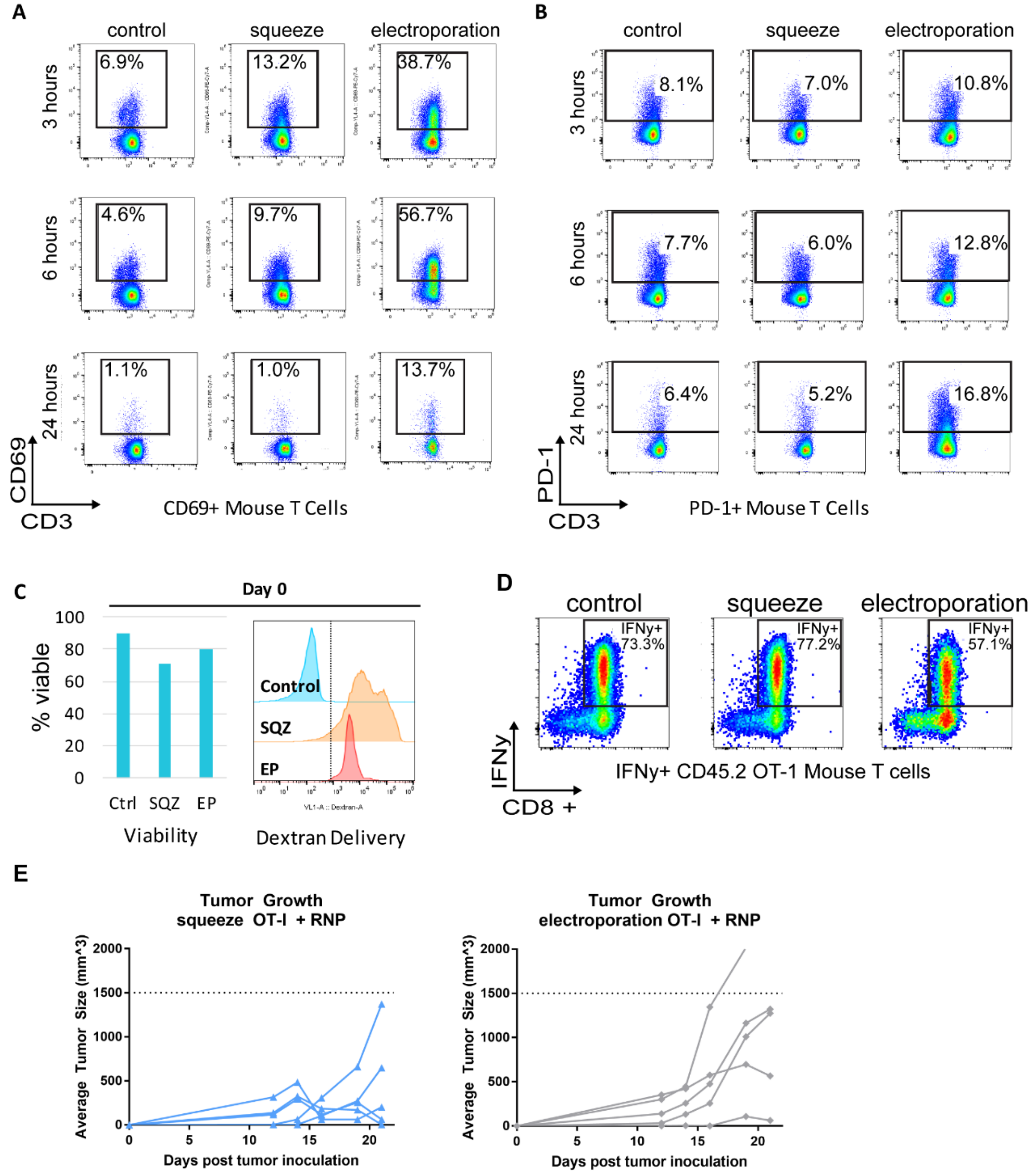

Supplementary Figure 3: *In vitro* and *in vivo* T cell functional flow cytometry results. Gating strategy for assessment of A) CD69 and B) PD-1 activation in CD3+ murine T cells treated with 3kDa dextran. Dextran was delivered via squeezing (SQZ), or electroporation (EP). Samples were measured at 3, 6, or 24 hours post-delivery. C) Cell viability and delivery were measured in a small subset of cells during the in vivo assessment of treatment-mediated T cell function assay to establish that treatment parameters would deliver material and would not adversely affect cell health. D) Gating strategy for assessment of IFNy activation in CD45.2+/CD8+ murine OT-I T cells. E) Tumor growth curves for individual mice are shown.
